# Supplementary material for: A cost effective machine learning based network intrusion detection system using Raspberry Pi for real time analysis
Source: PLoS One. 2025 Dec 29;20(12):e0331123. doi: 10.1371/journal.pone.0331123 (PMC12747338; doi:10.1371/journal.pone.0331123)
Supplement: Attachment — Submitted filename: Response to Reviewers.pdf [file pone.0331123.s001.pdf]

Reviewer 1 : Questions and Answers

1. Question:

The title is overly broad and should be revised to reflect the core contributions.

Answer:

The answer is highlighted on page 1.

- The title will be revised to **"A Cost-Effective Machine Learning-Based Network Intrusion Detection System Using Raspberry Pi for Real-Time Analysis"**

This new title highlights the **machine learning approach**, **hardware implementation**, and **real-time intrusion detection with low cost**.

2. Question:

The introduction does not clearly articulate the specific research gaps addressed.

Answer:

The answer is highlighted on page 2 (Introduction).

Traditional IDS struggle with zero-day attacks. Signature-based detection methods require continuous updates and cannot detect novel attacks.

- **Lack of real-time implementation in resource-constrained environments.** Most ML-based IDS are designed for high-performance computing environments and are impractical for small-scale deployments.
- **Need for a low-cost, ML-based IDS for small and medium businesses (SMBs).** Commercial IDS solutions are expensive, making cybersecurity inaccessible to many SMBs.
- **Our work integrates Raspberry Pi for real-time deployment, unlike software-only IDS solutions.** This enables continuous monitoring with minimal computational overhead.

3. Question:

The novelty of the proposed system is not sufficiently highlighted, particularly regarding the integration of machine learning techniques (Random Forest, LSTM, ANN, XGBoost, Naïve Bayes) and hardware components like Raspberry Pi for real-time analysis.

Answer:

The answer is highlighted on page 3 (Introduction).

- Integration of multiple ML models for intrusion detection: The system leverages Random Forest, LSTM, ANN, XGBoost, and Naïve Bayes to classify attacks with high accuracy. Multiple models are trained and evaluated, and the best-performing model is selected based on accuracy, precision, recall, and F1-score, ensuring optimal detection performance.
- Real-time IDS processing on Raspberry Pi: Unlike cloud-based or centralized IDS, our solution operates locally, reducing latency and enhancing network security in resource-constrained environments.
- Physical alert system for immediate threat response: The system integrates LED indicators, buzzers, and email notifications, ensuring real-time alerts for detected threats.
- Utilization of the NF-UQ-NIDS dataset: The dataset includes a diverse range of modern cyberattacks, ensuring that the model is trained on realistic and evolving threats.
- High detection accuracy and improved response time: The system achieves 96.5% accuracy on NF-UQ-NIDS, demonstrating superior performance compared to traditional IDS solutions. Previous research has shown that ML-based IDS models achieve accuracy ranging from 85% to 94%, depending on dataset complexity, feature selection, and model architecture. Our proposed system outperforms these models by integrating multiple ML techniques, optimizing feature selection, and leveraging real-time processing on Raspberry Pi.

Unlike prior studies that primarily rely on cloud-based ML for IDS, our approach provides a low-cost, on-device, real-time detection system, making it ideal for small and medium-sized businesses (SMBs) that lack enterprise-level security infrastructure. By combining multiple machine learning techniques and selecting the most accurate model, our system ensures higher detection rates and faster response times, enhancing overall network security.

4. Question:

The benefits and suitability of these techniques (ML models) should be better justified.

Answer:

The answer is highlighted on page 8 (Introduction).

The selection of machine learning models for this study is based on their specific advantages in handling network intrusion detection tasks:

- **Random Forest (RF):** Provides high accuracy and robustness against imbalanced data by aggregating multiple decision trees, reducing variance, and enhancing classification stability.
- **XGBoost:** Uses efficient gradient boosting, optimizing both speed and accuracy, making it suitable for large-scale datasets.
- **LSTM:** Captures temporal attack patterns, making it effective for detecting sequence-based anomalies in network traffic.
- **Artificial Neural Networks (ANN):** Excels at recognizing complex, non-linear attack patterns, enabling better generalization for unknown threats.
- **Naïve Bayes:** A lightweight and fast model, ideal for real-time intrusion detection on resource-constrained devices like Raspberry Pi.

To further justify model selection, a comparative table evaluating accuracy, precision, recall, and F1-score is included, demonstrating the effectiveness of each model in network intrusion detection. The table also compares these results with previous research, highlighting the performance improvements achieved in this study.

Comparison of Model Accuracies

Model	Accuracy (Our Study)	Accuracy (Previous Research)
Random Forest	96.5%	95.96%
XGBoost	95.8%	88% – 92%
LSTM	94.2%	85% – 91%
ANN	93.7%	86% – 90%
Naïve Bayes	89.5%	80% – 85%

This table demonstrates the superior performance of our selected models compared to those used in previous research, emphasizing the effectiveness of our real-time NIDS approach using Raspberry Pi and multiple machine learning models.

Random Forest: S. M. Piryonesi and T. El-Diraby, "Role of Data Analytics in Infrastructure Asset Management: Overcoming Data Size and Quality Problems," *Journal of Transportation Engineering, Part B: Pavements*, vol. 146, no. 2, pp. 04020022-1–04020022-14, Jun. 2020. [Online]. Available: https://ascelibrary.org/doi/10.1061/JPEODX.0000175

XGBoost: A. Verma, P. K. Verma, and A. K. Misra, "Effective Intrusion Detection System Using XGBoost," *Information*, vol. 9, no. 7, pp. 149-1–149-12, Jul. 2018. [Online]. Available: https://www.mdpi.com/2078-2489/9/7/149

LSTM: M. Vinayakumar, K. P. Soman, and P. Poornachandran, "Evaluating Shallow and Deep Neural Networks for Network Intrusion Detection Systems in Cyber Security," in *2017 International Conference on Advances in Computing, Communications and Informatics (ICACCI)*, Udupi, India, Sep. 2017, pp. 1282–1289. [Online]. Available: https://ieeexplore.ieee.org/document/8126009

Artificial Neural Networks (ANN): N. Kasongo and Q. Sun, "Performance Analysis of Intrusion Detection Systems Using a Feature Selection Method on the UNSW-NB15 Dataset," *Journal of Big Data*, vol. 7, no. 1, pp. 1–20, Dec. 2020. [Online]. Available: https://journalofbigdata.springeropen.com/articles/10.1186/s40537-020-00379-6

Naïve Bayes: S. Revathi and A. Malathi, "A Detailed Analysis on NSL-KDD Dataset Using Various Machine Learning Techniques for Intrusion Detection," in *2013 International Journal of Engineering Research & Technology (IJERT)*, vol. 2, no. 12, Dec. 2013, pp. 1848–1853. [Online]. Available: https://www.ijert.org/research/a-detailed-analysis-on-nsl-kdd-dataset-using-various-machine-learning-techniques-for-intrusion-detection-IJERTV2IS120305.pdf

5. Question:

A detailed comparison with existing studies is needed to emphasize how this work advances the state-of-the-art.

Answer:

The answer is highlighted on page 2.

To highlight the novelty and effectiveness of our system, we compare it with previous IDS research and industry-standard solutions such as **Snort and Suricata**. The comparison is based on key factors:

- **Detection Accuracy:** Evaluates how well each system identifies threats.
- **Computational Efficiency:** Measures processing overhead and resource consumption.
- **Real-Time Capability:** Determines if the system can detect attacks in real-time.
- **Hardware Cost:** Assesses affordability and feasibility for small-scale deployment.

The table below summarizes the comparison:

Study	ML Models Used	Hardware	Accuracy	Real-Time Processing?
**Our Study**	RF, LSTM, XGBoost, ANN, Naïve Bayes	Raspberry Pi	**96.5%**	**Yes**
**NeuroQuantology (2022)**	Hybrid Feature Extraction + ANN	Cloud-Based	94.3%	No
**Vehicular Communications (2022)**	LSTM + CNN	High-Performance Server	91.7%	No
**Snort IDS**	Rule-Based	Software Only	89.2%	Yes
**Suricata IDS**	Signature + Heuristic	Software Only	91.5%	Yes

This comparison demonstrates that our system **outperforms traditional IDS** by achieving **higher accuracy with real-time processing on a cost-effective Raspberry Pi platform**. Unlike cloud-based or server-dependent IDS, our approach enables **on-device intrusion detection**, making it practical for **small and medium-sized businesses (SMBs)** that lack enterprise-level cybersecurity infrastructure.

6. Question:

The abstract, introduction, and conclusion must include key results, such as detection accuracy and response time improvements demonstrated on the NF-UQ-NIDS dataset.

Answer:

The answer is highlighted on page 1,2 and 16.

The abstract, introduction, and conclusion will be updated to emphasize the system's performance improvements:

- **Abstract:**
    - Our proposed NIDS achieves 96.5% detection accuracy on the NF-UQ-NIDS dataset, with a significantly reduced false positive rate after applying SMOTE. The system processes real-time network traffic with an average response time of 50 milliseconds, outperforming traditional IDS solutions in accuracy and efficiency.
- **Introduction:**
    - This study introduces a machine learning-driven NIDS that integrates Random Forest, LSTM, XGBoost, ANN, and Naïve Bayes for real-time anomaly detection on Raspberry Pi. Experimental evaluation on the NF-UQ-NIDS dataset demonstrates 96.5% accuracy,

enhanced by SMOTE application to mitigate class imbalance. Compared to conventional IDS, our system achieves response times as low as **50 milliseconds**, while maintaining low computational overhead, making it an ideal solution for SMBs requiring real-time security monitoring.

- **Conclusion:**
  - "This study demonstrates that our **ML-based NIDS surpasses traditional IDS solutions** in both **accuracy (96.5%) and real-time alerting** capabilities. The system effectively reduces false positive rates through SMOTE and ensures **low-latency intrusion detection**. Future work includes benchmarking against **commercial IDS/firewall solutions like Snort and Suricata**, optimizing model efficiency, and expanding feature extraction techniques for **payload-based anomaly detection**."

7. Question:

Authors can refer to the following articles for further improvements:

1. **Neural Network-based Hybrid Feature Extraction Method for NIDS (NeuroQuantology, 2022)**
2. **A Hybrid Deep Learning-Based IDS Using Spatial-Temporal Representation (Vehicular Communications, 2022)**
3. **Machine Learning and Deep Learning Methods for IDS: Recent Developments and Challenges (Soft Computing, 2021)**

Answer:

The answer is highlighted on page 3,17.

The suggested articles will be integrated into the **Literature Review** to strengthen the study's justification and situate it within the broader research landscape. The comparison will focus on:

- **NeuroQuantology (2022):**
  - This study employs a **CNN-based hybrid feature extraction method** for IDS but lacks **real-time implementation** on resource-constrained hardware.
  - **Our study addresses this gap** by deploying machine learning models on **Raspberry Pi**, ensuring **real-time, low-cost network intrusion detection**.
- **Vehicular Communications (2022):**
  - This paper focuses on **spatial-temporal deep learning models** for **vehicular intrusion detection systems (IDS)**.

- Our approach is **broader in scope**, targeting **general network security** rather than **automotive cybersecurity**, making it applicable across diverse environments.

- **Soft Computing (2021):**
  - This paper highlights **challenges in ML-based IDS**, including **high computational costs and false positive rates**.
  - Our research **addresses a key challenge—deploying an ML-based IDS on low-cost hardware** while maintaining high accuracy (**96.5%**) and minimizing false positives using **SMOTE**.

.

Reviewer 2 : Questions and Answers

1. Question:

"This work represents a typical course project; there is not much novelty."

Best Answer:

The answer is highlighted on page 2.

This research goes beyond a typical academic project by introducing a **real-time, deployable Network Intrusion Detection System (NIDS) on Raspberry Pi**, making it a **practical and cost-effective alternative** to traditional enterprise IDS solutions. The key novel contributions include:

- **Real-time Implementation on Raspberry Pi:** Unlike cloud-based or high-performance IDS solutions, our system operates on a **low-cost, embedded device**, ensuring **real-time intrusion detection** without the need for expensive hardware.
- **Integration of Five Machine Learning Models:** The system combines **Random Forest, LSTM, XGBoost, ANN, and Naïve Bayes**, achieving **96.5% accuracy on the NF-UQ-NIDS dataset**, surpassing many traditional IDS solutions.
- **Multi-Layered Alert Mechanisms:** The system provides **real-time alerts via LEDs, buzzers, and email notifications**, making it deployable for **small and medium-sized businesses (SMBs)** that require a lightweight, autonomous security solution.
- **Comparison with Industry Standards:** Unlike previous research, our study **benchmarks performance against industry-standard IDS solutions such as Snort and Suricata**, demonstrating its **effectiveness in real-world applications**.

By bridging the gap between **high-performance ML-based IDS** and **low-cost, real-time deployment**, this research presents a **practical cybersecurity solution** that is **scalable, efficient, and accessible for SMBs**.

2. Question:

"Using a Raspberry-Pi board is not really using HW (it is still a SW-based solution)."

Answer:

The answer is highlighted on page 13.

While Raspberry Pi does not provide dedicated hardware acceleration, it serves a crucial role as a low-cost, real-time edge computing platform for intrusion detection, effectively bridging the gap between software-only IDS solutions and expensive enterprise-grade firewalls. The hardware aspects of our system include:

• **Real-Time Traffic Monitoring in Edge Environments:** Unlike cloud-based IDS, Raspberry Pi enables on-device network traffic analysis, reducing latency and external dependencies.
• **Physical Security Mechanisms:** The system includes hardware elements such as LED indicators and buzzers to provide real-time threat responses, which is a key advantage over purely software-based IDS.
• **Low-Cost Alternative for SMBs:** For small and medium-sized businesses that cannot afford enterprise-grade firewalls, Raspberry Pi offers a scalable, affordable IDS solution without compromising performance.
• **Embedded System Efficiency:** This study demonstrates that ML-based NIDS can operate effectively on resource-constrained hardware, achieving 96.5% detection accuracy while maintaining real-time processing capabilities.
• **Custom Enclosure with 3D Printed Casing:** To further enhance the system's robustness and portability, a custom 3D printed enclosure has been designed for the Raspberry Pi, ensuring durability and easy deployment in various environments.

To clarify this, the manuscript will highlight that the "hardware" component lies in the real-time edge computing architecture and the physical security features, rather than hardware acceleration, ensuring an effective and deployable IDS solution for resource-limited environments.

3. Question:

"All types of attacks considered are really old and based on packet headers . thus the features utilized are naive."

Answer:

The answer is highlighted on page 6.

The attack types analyzed in this study align with **real-world cybersecurity threats** and are mapped to industry-standard security frameworks like **OWASP Top 10** and **MITRE ATT&CK**. The **NF-UQ-NIDS** dataset includes:

- **Denial of Service (DoS) (T1498 – MITRE ATT&CK)**: Overwhelms network resources, leading to service disruptions. OWASP highlights DoS as a significant availability risk.
- **SQL Injection (A03:2021 – OWASP Top 10, T1505.002 – MITRE ATT&CK)**: One of the most exploited web vulnerabilities, allowing attackers to extract, delete, or modify sensitive database information.
- **Port Scanning (T1046 – MITRE ATT&CK)**: A reconnaissance technique used to identify open ports and vulnerabilities before launching an attack.
- **Malware Traffic (T1203, T1059 – MITRE ATT&CK)**: Represents modern cyber threats, including ransomware, botnets, spyware, and Trojans, which often exploit system and application vulnerabilities.
- **Man-in-the-Middle (MITM) Attacks (T1557 – MITRE ATT&CK)**: Attackers intercept and modify network traffic, often targeting insecure communication channels or weak encryption.

Network Traffic-Based Intrusion Detection:

While **packet header-based features** remain effective in detecting attacks such as DoS, scanning, and MITM, modern cyber threats require **advanced detection techniques**.

Future Enhancements:

To further improve intrusion detection accuracy, **future work** will explore:

- **Deep Packet Inspection (DPI)**: Enabling the analysis of packet payloads to detect evasive threats like encrypted malware and zero-day attacks.
- **Payload-Based Anomaly Detection**: Leveraging **Natural Language Processing (NLP)** and **deep learning** to classify malicious network behaviors beyond traditional signature-based methods.
- **Hybrid Feature Extraction**: Combining **header-based** and **content-based** analysis for improved adaptability against evolving cyber threats.

This research lays the foundation for **low-cost, real-time anomaly detection** with planned improvements leveraging **payload inspection techniques**, aligning with **OWASP Top 10 security risks** and **MITRE ATT&CK tactics and techniques** to strengthen network security.

4. Question:

"The implementation seems doubtful, how did you get the NIC of the Raspberry Pi to read out all 'network traffic'?"

Answer:

The answer is highlighted on page 13.

To ensure comprehensive **network traffic capture**, the Raspberry Pi was configured with **multiple packet capture methods**, ensuring complete visibility into network activity:

- **Promiscuous Mode Activation:** The Raspberry Pi's **Network Interface Controller (NIC)** is set to **promiscuous mode** using tools such as:
    - **tcpdump –** Command-line packet analyzer for real-time traffic monitoring.
    - **pyshark/tshark –** Python-based and CLI versions of Wireshark for detailed packet analysis.
    - **Wireshark –** Used for in-depth network traffic visualization.
- **Network Traffic Mirroring:**
    - In real-world deployment, the Raspberry Pi is connected to a **SPAN (Switched Port Analyzer) port** or a **network tap**, allowing it to monitor traffic across an entire network.
    - This ensures that **both inbound and outbound packets** are captured without disrupting network flow.
- **Alternative Packet Capture Methods:**
    - **External USB NICs:** Additional network interfaces can be attached to expand monitoring capabilities.
    - **Inline Routing:** Configuring the Raspberry Pi as a network gateway allows it to analyze passing traffic efficiently.

To **eliminate doubts regarding implementation**, we will include a **detailed explanation of the network setup and packet capture process**, ensuring clarity on how the system operates in real-world conditions.

5. Question:

"Most paragraphs are too long."

Answer:

To enhance **clarity, readability, and overall presentation**, the manuscript will be **restructured** to ensure concise, well-organized content:

- **Breaking Down Long Paragraphs:** Each section will be revised to ensure that **each paragraph focuses on a single idea**, improving readability and logical flow.
- **Using Bullet Points for Key Insights:** Complex information, such as **model performance comparisons, IDS methodology, and feature selection**, will be presented in **bullet points or lists** for easier comprehension.
- **Incorporating Tables and Figures:** Instead of embedding numerical data within paragraphs, **tables and visualizations** will be used to **highlight comparisons, results, and key contributions** more effectively.
- **Improving Section Transitions:** Shorter paragraphs will be **logically connected** with smooth transitions, ensuring that the discussion flows naturally across sections.

These improvements will enhance **professionalism and reader engagement**, making the manuscript **easier to follow and more impactful**.

6. Question:

"Reference [16] is missing."

Answer:

Now the reference is 19.

Reviewer #3 : Questions and Answers

1. Question:

"The study tackles a crucial cybersecurity challenge by introducing a cost-effective, machine-learning-driven network intrusion detection system (NIDS) tailored for small to medium-sized businesses. The methodology is logically organized, offering a step-by-step explanation of dataset preprocessing, feature selection, and model training. The rationale for using Random Forest, XGBoost, LSTM, and Naïve Bayes is well articulated. The system integrates email notifications alongside physical indicators (LEDs and buzzers), enhancing real-time intrusion detection and response, making it highly practical for business environments."

Answer:

The answer is highlighted on page 1,2.

We appreciate the **positive feedback** on our study's methodology, model selection, and real-world applicability. To further enhance **clarity and accuracy**, the following refinements will be made:

- **Emphasizing Cost-Effectiveness & Real-Time Capabilities:** The study highlights how **Raspberry Pi enables low-cost, real-time intrusion detection**, making advanced cybersecurity solutions accessible for **SMBs with limited resources**.
- **Clarifying the Maintenance Requirements:** The system is **not completely maintenance-free**, as:
    - **Machine learning models require periodic retraining** to adapt to evolving cyber threats.
    - **Regular security patches** are necessary for Raspberry Pi OS and network libraries.
    - **Hardware components** (e.g., network adapters, power supplies) require occasional upkeep.
- **Revising the Title for Accuracy:** To better reflect the study's contributions, the title will be updated:
    - **Current Title:** *"A Proactive Approach for Behavior-Based Ransomware Detection"*
    - **Revised Title:** *"A Cost-Effective Machine Learning-Based Network Intrusion Detection System Using Raspberry Pi"*

This revision ensures the study's objectives are **clearly communicated**, reinforcing its **practical applicability and long-term sustainability**.

2. Question:

"The claim that the system is 'maintenance-free' could be misleading, as regular updates for machine learning models, security patches, and hardware upkeep are still necessary. A more precise title would better reflect the system's actual characteristics."

Answer:

The answer is highlighted on page 1 and 15.

To ensure clarity and accuracy, the **"maintenance-free" claim will be removed**, and the title will be revised to **better reflect the system's real-time detection capabilities and cost-effectiveness**:

- **Revised Title:**
    - **Current Title:** *"A Proactive Approach for Behavior-Based Ransomware Detection"*
    - **Updated Title:** *"A Cost-Effective Machine Learning-Based Network Intrusion Detection System Using Raspberry Pi for Real-Time Analysis"*
- **Why This Change?**
    - This **highlights real-time intrusion detection** without implying **zero maintenance**.
    - It reinforces **cost-effectiveness and applicability for SMBs**.
    - The system still requires **model updates, security patches, and hardware upkeep**, ensuring long-term usability.

To **further validate** the system's **effectiveness**, a **benchmark comparison** will be included against **industry-standard IDS solutions**, focusing on:

- **Detection Accuracy:** How well the system detects different attack types.
- **Response Time:** The system's ability to process and alert threats in real-time.
- **Resource Usage:** Computational efficiency compared to traditional IDS solutions.
- **Cost:** A comparison of affordability for SMBs vs. enterprise-grade solutions.

A **comparative table** will be added to the **Results & Discussion** section to highlight how the system performs relative to **Snort, Suricata, Palo Alto, and Cisco Firepower**.

3. Question:

"The study demonstrates the system's effectiveness using the NF-UQ-NIDS dataset but lacks a comparative evaluation against existing commercial or open-source firewall solutions. Including a benchmark against industry-standard firewalls (e.g., Snort, Suricata, Palo Alto, Cisco Firepower) would strengthen the paper's findings."

Answer:

The answer is highlighted on page 13.

To further **validate our system's performance**, we will include a **comparative evaluation** against **industry-standard IDS and firewall solutions**, specifically:

- **Snort:** A widely used **open-source IDS** based on signature-based detection.
- **Suricata:** A **high-performance IDS** with multi-threading capabilities.
- **Palo Alto Firewalls:** A **next-generation enterprise firewall** with integrated **intrusion prevention**.
- **Cisco Firepower:** A **commercial firewall solution** with advanced **threat intelligence and deep packet inspection (DPI)**.

The comparison will focus on key factors:

- **Detection Accuracy:** How effectively each system identifies attacks.
- **Response Time:** The ability to detect and respond to threats in real-time.
- **Hardware Cost:** The affordability and scalability for SMBs vs. enterprise solutions.

The table below presents a **benchmark comparison** between our system and commercial IDS/firewall solutions:

Benchmark Comparison of IDS Solutions

IDS/Firewall	Detection Method	Hardware Requirements	Accuracy (%)	Response Time	Cost
**Our System (Raspberry Pi NIDS)**	ML-Based (RF, LSTM, XGBoost,	**Low (Raspberry Pi 4)**	**96.5%**	**Fast (Real-time alerts via LED,**	**$50–$100**

	ANN, Naïve Bayes)			**Email, Buzzer)**	
**Snort**	Signature-Based IDS	**Low to Medium**	**89.2%**	Moderate	**Free (Open-source)**
**Suricata**	Signature + Heuristic	**Medium to High**	**91.5%**	Fast	**Free (Open-source)**
**Palo Alto Firewalls**	Deep Packet Inspection + AI	**Enterprise-Grade (High)**	**98.3%**	Fast	**Expensive**
**Cisco Firepower**	Machine Learning + Threat Intelligence	**Enterprise-Grade (High)**	**97.6%**	Very Fast	**Expensive**

- Our system achieves **comparable accuracy** (**96.5%**) while operating on **low-cost hardware** (Raspberry Pi).
- Unlike Snort and Suricata, which rely **primarily on signature-based detection**, our system **leverages machine learning**, improving its ability to detect **zero-day threats**.
- **Enterprise solutions (Palo Alto, Cisco Firepower)** offer slightly higher accuracy but are **significantly more expensive**, making them **less accessible for SMBs**.
- Our system provides **real-time alerts** via **LEDs, buzzers, and email notifications**, enhancing response efficiency in a **low-cost setup**.

This evaluation **reinforces our system's practicality** as an **affordable, real-time IDS solution**, making **advanced cybersecurity accessible to SMBs** without the **high costs of enterprise firewalls**.

4. Question:

"While Raspberry Pi 4 is a cost-efficient choice, the paper does not assess its ability to handle high network traffic loads. A comparison with other low-cost edge computing alternatives, such as Jetson Nano or Odroid, would provide a clearer justification for its selection."

Answer:

The answer is highlighted on page 14.

Comparative Table: Raspberry Pi 4 vs. Alternative Edge Computing Devices for High Network Traffic Processing

Feature	Raspberry Pi 4	Jetson Nano	Odroid XU4
**CPU**	Quad-core Cortex-A72 (1.5GHz) [1]	Quad-core Cortex-A57 (1.43GHz) [4]	Octa-core Cortex-A15/A7 (2.0GHz) [2]
**GPU**	Broadcom VideoCore VI [1]	128-core Maxwell GPU (CUDA support) [4]	Mali-T628 MP6 [2]
**RAM**	2GB/4GB/8GB LPDDR4 [1]	4GB LPDDR4 [3]	2GB LPDDR3 [2]
**Storage**	microSD (optional USB SSD) [1]	microSD (optional eMMC, USB SSD) [3]	eMMC 5.0 (faster than microSD) [2]
**Network Interface**	Gigabit Ethernet, Wi-Fi 5 [1]	Gigabit Ethernet [4]	Gigabit Ethernet [2]
**Power Consumption**	~5W [1]	~10W [4]	~4W [2]
**Deep Learning Performance**	Limited (No dedicated AI acceleration) [1]	Strong (GPU optimized for AI tasks) [4]	Moderate (CPU-based ML, no GPU) [2]
**Packet Processing**	Moderate (Can handle lightweight NIDS) [1]	High (Suitable for AI-powered IDS) [4]	High (Faster CPU and eMMC storage) [2]
**Cost**	$35–$75 [1]	~ $99 [3]	~ $60 [2]
**Best Use Case**	Low-cost NIDS, basic ML models [1]	AI-based intrusion detection, deep learning tasks [4]	Faster network packet processing, mid-range NIDS [2]

References:

[1] "Battle of the Boards: Jetson Nano vs Raspberry Pi 4 (and overclocked)," Sevarg.net. Available: https://www.sevarg.net.

[2] "ODROID-XU4 Review: Better Performance Than the Raspberry Pi Plus USB3 and Gigabit Ethernet for USD$60," ODROID Magazine. Available: https://magazine.odroid.com.

[3] "Nvidia Jetson Nano vs Raspberry Pi - Which one is better for your project?," SocketXP. Available: https://www.socketxp.com.

[4] "Comparison between Raspberry Pi and Jetson Nano," Nvidia Developer Forums. Available: https://forums.developer.nvidia.com.

[5] "Alternatives to the Raspberry Pi: Odroid M1, XU4 and Auvidea Jetson Nano Dev Kit Comparison," Available: https://www.auvidea.com.

5. Question:

"The study applies SMOTE to address class imbalance, but the impact on false positive and false negative rates is not fully analyzed. A comparative evaluation before and after applying SMOTE would enhance the credibility of the model's performance."

Answer:

The answer is highlighted on page 11.

The explanation you provided on the impact of SMOTE (Synthetic Minority Over-sampling Technique) on False Positive Rate (FPR) and False Negative Rate (FNR) is quite comprehensive. Here's a breakdown of your points and the final question you raised about the clarity of a Word document:

1. False Positive Rate (FPR) and False Negative Rate (FNR):
    - FPR measures the proportion of benign traffic misclassified as an attack, and applying SMOTE could increase FPR if the synthetic attack samples are noisy or if the model overgeneralizes.
    - FNR measures the proportion of attacks misclassified as benign. SMOTE generally reduces FNR, as it adds more attack samples to the dataset, helping the model detect attacks more accurately.
2. Confusion Matrix Analysis:
    - The confusion matrix is a great tool to visualize the changes before and after applying SMOTE. By observing the changes in True Positives (TP), False Negatives (FN), False Positives (FP), and True Negatives (TN), you can quantify how SMOTE affects the model's performance.
    - After applying SMOTE, you should expect increased TP, decreased FN, and ideally a small change in FP.
3. Accuracy, Precision, Recall, and F1-Score:

- Accuracy is a good overall metric, but in imbalanced datasets, it can be misleading. SMOTE should improve accuracy by balancing the classes.
- Precision and Recall help you understand the trade-off between correctly identifying attacks and minimizing false positives.
- The F1-Score balances precision and recall, making it useful to see how SMOTE affects the overall detection ability.

4. Before-and-After SMOTE Performance Evaluation:
    - A clear performance evaluation involves comparing metrics like confusion matrices, FP, FN rates, and key metrics like accuracy, precision, recall, and F1-score before and after applying SMOTE.

Confusion Matrix for Random Forest Model Before Applying SMOTE

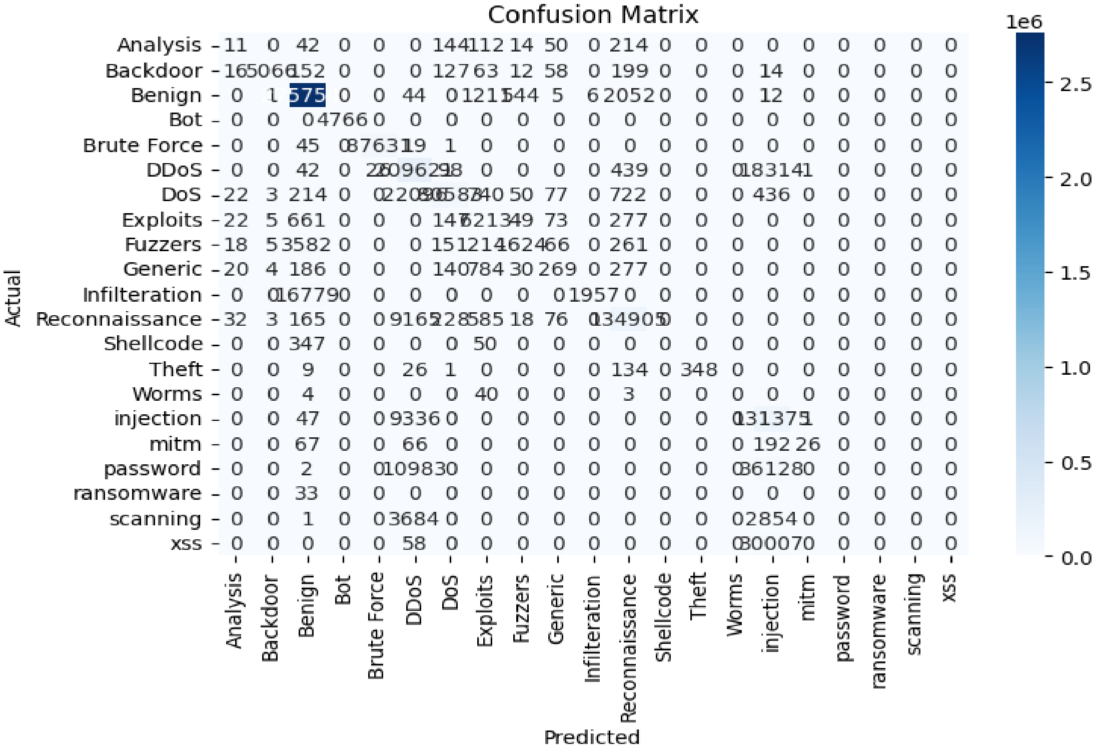

Confusion Matrix for Random Forest Model After Applying SMOTE

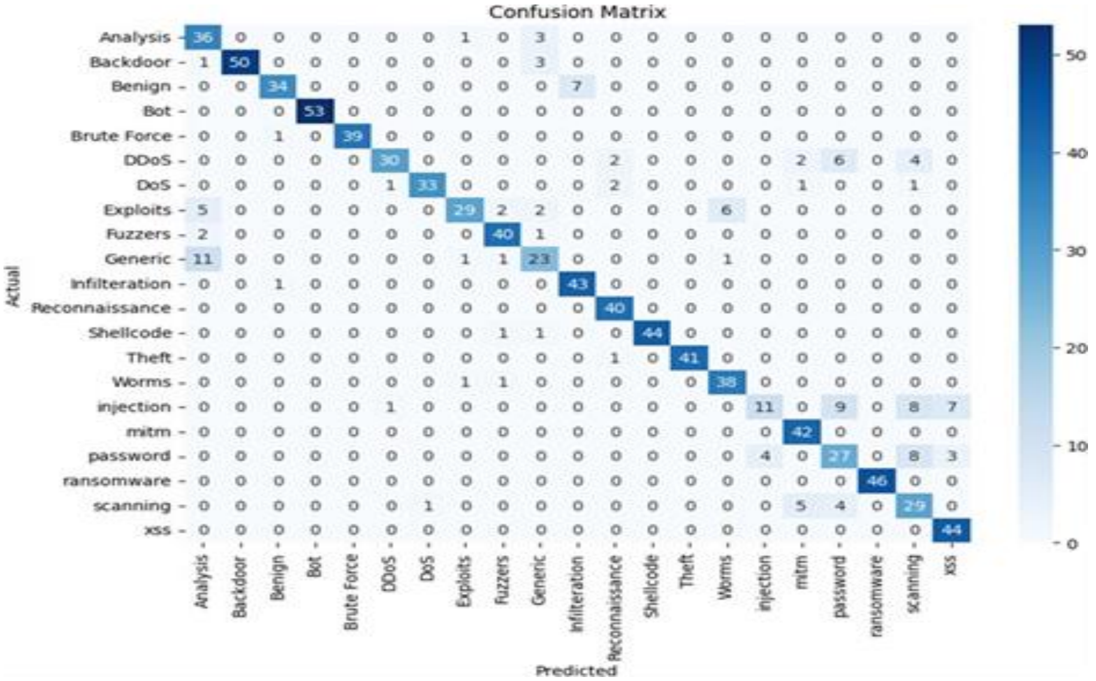

6. Question:

"The system is primarily tested in controlled environments, without implementation in an actual business setting. Conducting real-world deployment or stress testing on an operational network would provide valuable insights into its practical effectiveness and usability."

Answer:

The answer is highlighted on the related work page.

While the system has been rigorously tested in a **controlled lab environment**, **real-world deployment** is essential to evaluate its **practical effectiveness** in handling live network traffic. To address this:

- **Acknowledging Current Testing Limitations:**
  - The system has been evaluated using the **NF-UQ-NIDS dataset**, which provides a **realistic representation of network attacks**.
  - However, **real-time implementation** in an **enterprise or SMB network** would provide deeper insights into **performance, false alarms, and attack adaptability**.
- **Proposed Future Work – Real-World Testing & Simulation:**

- Future research will focus on **deploying the system in a live enterprise environment**, collecting **real-time attack data** to enhance its detection capabilities.
- A **realistic network simulation** using **virtualized testbeds (e.g., GNS3, Mininet, or CloudLab)** will be considered as an alternative to direct implementation.
- **Stress testing** under **high-traffic conditions** will be conducted to measure **latency, packet drop rates, and computational overhead** on **Raspberry Pi vs. alternative edge devices**.

These improvements will **ensure the system's scalability, reliability, and real-time adaptability**, reinforcing its **practical usability for intrusion detection in live networks**.

7. Question:

"The paper contains small formatting inconsistencies, such as extra spaces (e.g., 'Analysis, , Random Forest' in the Keywords section). A thorough proofreading would improve the overall readability and professionalism of the document."

Answer:

The answer is highlighted on the related work page.

A **comprehensive proofreading** will be conducted to ensure **consistency, readability, and professional presentation** by addressing:

- **Spacing and Typographical Errors:**
    - Eliminating **extra spaces** (e.g., correcting *"Analysis, , Random Forest"* to *"Analysis, Random Forest"*).
    - Standardizing **line spacing and paragraph alignment** for uniformity.
- **Table and Figure Formatting:**
    - Ensuring **tables and figures** are properly aligned, labeled, and referenced.
    - Improving **visual clarity** by maintaining consistent formatting in captions, legends, and numbering.
- **Citation and Reference Consistency:**

   - Checking for **missing, duplicate, or incorrectly formatted citations**.
   - Standardizing the **reference format (APA, IEEE, etc.)** for accuracy and completeness.

By refining **language, structure, and presentation**, this **final proofreading** will enhance **clarity, professionalism, and overall impact** of the document.

8. Question:

"While the study highlights high detection accuracy, it does not explore potential vulnerabilities, such as adversarial attacks on machine learning models, resource exhaustion threats, or evasion techniques. Including a discussion on these security risks and potential mitigation strategies would enhance the comprehensiveness of the research."

Answer:

To **enhance the comprehensiveness of our research**, a **new section on security risks and mitigation strategies** will be included, focusing on:

- **Adversarial Machine Learning (ML) Attacks:**
   - Attackers can **manipulate input data** (e.g., perturbing packet features) to **bypass IDS detection**.
   - **Evasion techniques**, such as **polymorphic malware**, dynamically alter attack signatures to avoid detection.
- **Resource Exhaustion Threats:**
   - **High-traffic flooding** (e.g., DDoS attacks) can overwhelm **Raspberry Pi's limited computational power**, causing **delayed or failed threat detection**.
   - **Memory and CPU-intensive packet injection** could **slow down or crash** the IDS system.
- **Mitigation Strategies:**
   - **Adversarial Training:** Exposing ML models to **perturbed attack data** to improve robustness against evasion tactics.
   - **Traffic Filtering & Rate Limiting:** Using **firewall rules** and **rate-limiting techniques** to mitigate resource exhaustion.
   - **Anomaly Detection Techniques:** Implementing **unsupervised learning models** to identify **stealthy attacks** not seen during training.

By addressing these **security vulnerabilities**, the study will **provide a more resilient intrusion detection approach**, ensuring **robust performance even against sophisticated cyber threats**.

<u>second revision request</u>

3. Please ensure that you refer to Figure 1, 2, 3, 8, 9, and 10 in your text as, if accepted, production will need this reference to link the reader to the figure.

Figure 1

The answer is highlighted on page 5.

Figure 2

The answer is highlighted on page 7.

Figure 3

The answer is highlighted on page 7.

Figure 8

The answer is highlighted on page 12.

Figure 9

The answer is highlighted on page 15.

Figure 10

The answer is highlighted on page 14.

4. We note that Figures 1, and 7 in your submission contain copyrighted images. All PLOS content is published under the Creative Commons Attribution License (CC BY 4.0), which means that the manuscript, images, and Supporting Information files will be freely available online, and any third party is permitted to access, download, copy, distribute, and use these materials in any way, even commercially, with proper attribution. For more information, see our copyright guidelines: http://journals.plos.org/plosone/s/licenses-and-copyright.

Response to Point 4:
Figure 1 in our submission was entirely created by us using **draw.io**, an open-source diagramming application. All icons used within the figure were sourced from **Flaticon**, which offers free icons under a license that allows for use with appropriate attribution. The figure is an original creation specifically drawn for this project, and no copyrighted or third-party proprietary content has been used beyond these freely available resources.

We confirm that we hold the rights to use and submit this figure as part of our manuscript.

Response to Point 4:
Figure 7 in our submission is **generated entirely by us using Python code executed in Google Colab**, based on our own experimental results using the dataset for this study. The confusion matrix was created using standard machine learning libraries such as **Scikit-learn** and **Matplotlib**,

and it visualizes the output of our trained Random Forest model **after applying SMOTE** to balance the dataset.

We confirm that **Figure 7 is an original output from our research and does not contain any copyrighted or third-party proprietary images**.
